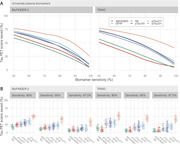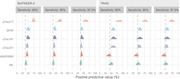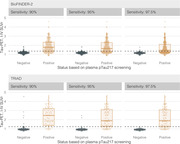# Optimizing tau‐PET referrals in memory clinics through a blood biomarker workflow

**DOI:** 10.1002/alz.093092

**Published:** 2025-01-09

**Authors:** Wagner Scheeren Brum, Nicholas Cullen, Joseph Therriault, Shorena Janelidze, Nesrine Rahmouni, Jenna Stevenson, Stijn Servaes, Andrea L. Benedet, Eduardo R. Zimmer, Erik Stomrud, Sebastian Palmqvist, Henrik Zetterberg, Giovanni Frisoni, Nicholas J. Ashton, Kaj Blennow, Niklas Mattsson‐Carlgren, Pedro Rosa‐Neto, Oskar Hansson

**Affiliations:** ^1^ Department of Psychiatry and Neurochemistry, Institute of Neuroscience and Physiology, The Sahlgrenska Academy, University of Gothenburg, Mölndal Sweden; ^2^ Graduate Program in Biological Sciences: Biochemistry, Universidade Federal do Rio Grande do Sul (UFRGS), Porto Alegre Brazil; ^3^ Clinical Memory Research Unit, Lund University, Malmö Sweden; ^4^ McGill University, Montreal, QC Canada; ^5^ Clinical Memory Research Unit, Lund University, Lund Sweden; ^6^ Translational Neuroimaging Laboratory, The McGill University Research Centre for Studies in Aging, Montréal, QC Canada; ^7^ Department of Psychiatry and Neurochemistry, Institute of Neuroscience and Physiology, The Sahlgrenska Academy, University of Gothenburg, Mölndal, Gothenburg Sweden; ^8^ Federal University of Rio Grande do Sul (UFRGS), Porto Alegre, RS Brazil; ^9^ Clinical Memory Research Unit, Department of Clinical Sciences Malmö, Faculty of Medicine, Lund University, Lund Sweden; ^10^ Clinical Memory Research Unit, Department of Clinical Sciences, Lund University, Lund Sweden; ^11^ University Hospitals and University of Geneva, Geneva Switzerland

## Abstract

**Background:**

Blood‐based biomarkers have demonstrated great promise for identifying biomarker‐confirmed Alzheimer's disease. We aimed to evaluate whether blood‐based biomarkers could optimize the referral of memory clinic patients to a tau‐PET exam, which is crucial for prognostic evaluation.

**Method:**

The study measured various plasma biomarkers (Aβ42/Aβ40, pTau181, pTau217, pTau231, NfL, and GFAP) and compared them with tau‐PET scan results in patients with subjective cognitive decline, mild cognitive impairment, or dementia. Participants were sourced from the Swedish BioFINDER‐2 study (548 individuals) and the TRIAD study (179 individuals). Cutoffs for each biomarker were established at 90%, 95%, and 97.5% sensitivity for detecting tau‐PET‐positivity. We then calculated the percentage of patients below these cutoffs (to potentially avoid unnecessary tau‐PET scans) and the tau‐PET‐positivity rate among those above the cutoffs.

**Result:**

Plasma pTau217 showed the most promising results. At a 95% sensitivity cutoff in both cohorts, using pTau217 could avoid nearly half of the tau‐PET scans while maintaining a tau‐PET‐positivity rate of approximately 70% in those referred. Furthermore, tau‐PET was strongly associated with subsequent cognitive decline. In the BioFINDER‐2 cohort, tau‐PET predicted cognitive decline only in individuals above the plasma pTau217 referral cutoff, suggesting a more targeted and informative use of tau‐PET scans.

**Conclusion:**

Plasma pTau217 demonstrates potential as a guiding biomarker for selecting Alzheimer’s disease patients for tau‐PET scans, particularly when accurate prognostic information is clinically valuable. This approach could lead to more efficient and informative use of tau‐PET scans, avoiding unnecessary procedures in patients unlikely to benefit from them.